# Single-cell RNA sequencing revealed subclonal heterogeneity and gene signatures of gemcitabine sensitivity in pancreatic cancer

**DOI:** 10.3389/fphar.2023.1193791

**Published:** 2023-06-01

**Authors:** Zelin Hou, Jiajing Lin, Yuan Ma, Haizhong Fang, Yuwei Wu, Zhijiang Chen, Xianchao Lin, Fengchun Lu, Shi Wen, Xunbin Yu, Heguang Huang, Yu Pan

**Affiliations:** ^1^ Department of General Surgery, Fujian Medical University Union Hospital, Fuzhou, China; ^2^ Department of Pathology, Fujian Provincial Hospital, Fuzhou, China

**Keywords:** pancreatic cancer, gemcitabine, therapy resistance, single-cell RNA sequencing, PDAC, pancreatic ductal adenocarcinoma

## Abstract

**Introduction:** Resistance to gemcitabine is common and critically limits its therapeutic efficacy in pancreatic ductal adenocarcinoma (PDAC).

**Methods:** We constructed 17 patient-derived xenograft (PDX) models from PDAC patient samples and identified the most notable responder to gemcitabine by screening the PDX sets *in vivo*. To analyze tumor evolution and microenvironmental changes pre- and post-chemotherapy, single-cell RNA sequencing (scRNA-seq) was performed.

**Results:** ScRNA-seq revealed that gemcitabine promoted the expansion of subclones associated with drug resistance and recruited macrophages related to tumor progression and metastasis. We further investigated the particular drug-resistant subclone and established a gemcitabine sensitivity gene panel (GSGP) (SLC46A1, PCSK1N, KRT7, CAV2, and LDHA), dividing PDAC patients into two groups to predict the overall survival (OS) in The Cancer Genome Atlas (TCGA) training dataset. The signature was successfully validated in three independent datasets. We also found that 5-GSGP predicted the sensitivity to gemcitabine in PDAC patients in the TCGA training dataset who were treated with gemcitabine.

**Discussion and conclusion:** Our study provides new insight into the natural selection of tumor cell subclones and remodeling of tumor microenvironment (TME) cells induced by gemcitabine. We revealed a specific drug resistance subclone, and based on the characteristics of this subclone, we constructed a GSGP that can robustly predict gemcitabine sensitivity and prognosis in pancreatic cancer, which provides a theoretical basis for individualized clinical treatment.

## Introduction

### Background

Pancreatic ductal adenocarcinoma (PDAC) is a deadly malignancy with an overall 5-year survival rate of only 11% (Siegel et al., 2022). Gemcitabine has been considered the standard treatment and has been widely used as a first-line drug for advanced PDAC in the past decade. Although only approximately 10%–23% of patients with advanced PDAC are sensitive to standard chemotherapy that improves survival (Conroy et al., 2011), clinically, some patients who respond well to gemcitabine initially develop resistance during treatment or relapse after chemotherapy. Recent studies have revealed that the occurrence of drug resistance in tumors may be related to pre-existing drug-resistant cell subsets. Bhang et al. demonstrated that rare subpopulations resistant to small-molecule inhibitors and conventional chemotherapy may pre-exist in tumors prior to drug therapy in non-small-cell lung cancer (Bhang et al., 2015). Lee et al. reported that resistance to cytotoxic chemotherapy in bladder cancer was associated with pre-existing subpopulations (Lee et al., 2020). Seth et al. demonstrated that the pre-existing tumorigenic compartment functional heterogeneity was the origin of chemoresistance in pancreatic tumors (Seth et al., 2019). However, the tumor heterogeneity of PDAC has not been comprehensively studied.

Single-cell RNA sequencing (scRNA-seq) has greatly expanded the ability to explore tumor heterogeneity, and it has a high resolution for identifying rare tumor subgroups. In recent years, scRNA-seq has been used in the study of chemotherapy resistance in lung cancer, breast cancer, bladder cancer, and melanoma (Ding et al., 2020; Gregorc et al., 2021; Li et al., 2022; Wei et al., 2022). Some researchers have used this technology to study PDAC drug resistance, but studies on gemcitabine resistance in PDAC are still limited.

To investigate the remodeling of cell subpopulations in PDAC tumor tissues after gemcitabine chemotherapy, we constructed a patient-derived xenograft (PDX) model from PDAC patient samples that simulated the tumor response to chemotherapy and obtained tumor tissue samples pre- and post-chemotherapy. By screening the PDX sets *in vivo*, we identified responders and further investigated the most sensitive responder to gemcitabine. Using scRNA-seq, we revealed a specific drug-resistant subclone that existed before treatment and constructed a gemcitabine sensitivity gene panel (GSGP) based on the characteristics of this subclone to predict gemcitabine sensitivity and prognosis in PDAC.

## Materials and methods

### Patient and tissue samples

This study was carried out in accordance with the principles of the Declaration of Helsinki and approved by the Committee for the Ethical Review of Research, Fujian Medical University Union Hospital. Informed consent was obtained before sample collection. Surgical specimens of human pancreatic cancer were collected from the patients who received surgery at Fujian Medical University Union Hospital, Fuzhou, China, from January 2018 to January 2020. All patients had pathologic confirmation of PDAC. Patients with neoadjuvant treatment, inflammatory diseases, or active infection were excluded. The stage of each patient was assessed based on the American Joint Committee on Cancer version 8.

### Immunohistochemistry

The protocol of IHC was as described previously (Hou et al., 2021). The rabbit anti-CD68 antibody (ab283654, Abcam), anti-LY6G antibody (ab238132, Abcam), and rabbit anti-CCR2 antibody (ab273050, Abcam) were used for immunohistochemistry staining. The density of the positive cells was calculated with ×400 magnification in five representative fields in the tumor tissues, and the average was calculated. Each section was evaluated by two pathologists in a blinded manner.

### Patient-derived xenograft generation

PDX modeling was performed as described previously (Keysar et al., 2013; Olson et al., 2018). PDX models were generated from fresh tumor specimens of PDAC patients, which were collected from surgical specimens at Fujian Medical University Union Hospital. The fresh tumor tissue was cut into 0.3 × 0.3 × 0.3-cm pieces and then placed in RPMI supplemented with 10% fetal bovine serum at 4–8°C. The NOD-SCID mice were sterilized and anesthetized, and then the tumor pieces were directly inserted into the subcutaneous space of the flank. Mice were monitored and euthanized when the tumor reached 1,000 mm^3^. Subcutaneous tumors were removed and transplanted into a secondary colony of mice for PDX model maintenance. PDX models were maintained *in vivo* by serial transplantation of tumors, and all analyses performed to date were from passages ≤4.

### Mice

Mice used for the establishment of PDX models were 6–8-week-old male NOD-SCID (NOD/ShiLtJGpt-Prkdc^em26Cd52^/Gpt) mice, which were obtained from Nanjing Biomedical Research Institute of Nanjing University (Nanjing, China). Maintenance of PDX models and drug treatment studies were performed in the same mice. Animal experiment protocols were approved by the Ethics Committee for Animal Research of 900 Hospital of the Joint Logistics Team.

### *In vivo* drug screening

For the gemcitabine screen, 10–16 mice per model were engrafted with tumor fragments, and mice were divided into two groups randomly when subcutaneous tumor volume reached 150 mm^3^ (5–8 per group). Gemcitabine (Gemzar) was resuspended in saline and administered at 100 mg/kg twice per week by intraperitoneal injection, and control mice were treated with saline in the screen and validation studies. Tumor volume and body weight were monitored twice per week during the entire experimental period, and tumor volume was calculated by length × width (2 × 0.5). Tumor suppression activity was expressed as the relative tumor growth rate (T/C%) using the following formula: T/C% = (T_RTV_/C_RTV_)*100%, RTV = V_t_/V_0_ (T_RTV_ means the value of RTV of mice in the treatment group; C_RTV_ means the value of RTV of mice in the control group; RTV means relative tumor volume; V_0_ and V_t_ represent the tumor volume at initial time and final time for saline or gemcitabine treatment) (Yao et al., 2020). The 17 PDAC patients were grouped by T/C% less than 40% or T/C% more than 40% (Savage et al., 2017). One of the models demonstrated an exceptional response (PC-12), and a validation study was performed in randomized cohorts of six mice per arm. Mice were treated with gemcitabine or normal saline once the tumor volume reached 150 mm^3^ for 4 weeks. On day 28, after the first treatment, the mice were euthanized, and the tumors were harvested for weight and tumor volume measurements and immunohistochemical analyses for further studies. Tumors were also processed and digested for scRNA-seq.

### Tissue digestion and preparation for scRNA-seq

Fresh tumor tissues removed from the PDX model (PC-12) were placed in RPMI 1640 (Gibco) with 1% fetal bovine serum (Gibco) on ice to preserve viability. Tumor tissues were washed 2–3 times in phosphate-buffered saline and then cut into smaller pieces using sterile ophthalmic scissors on ice. Then, the pieces were transferred to a 15-mL centrifugal tube and digested using a tumor dissociation kit (Miltenyi Biotec). Tumor tissues were enzymatically digested at 37°C at a shaking speed of 50 r.p.m for 40–60 min. Dissociated cells were repeatedly collected at an interval of 20 minutes to increase the cell yield and viability. Then, the cell suspensions were filtered through 70-μm cell strainers (Miltenyi Biotec) and lysed in ACK lysis buffer (GS3309) to filter out cell clumps and remove red blood cells. Single cells were washed in PBS with 0.04% bovine serum albumin (Sigma). Dead cells were removed using the MACS Dead Cell Removal Kit (Miltenyi Biotec). Cells collected from each group (six replicate tumors were used in each group) were merged into one sample and resuspended in PBS with 0.04% BSA at a density of 1 × 10^6^ cells/ml in preparation for single-cell library creation. Cell viability and concentration were measured using the Countess II Automated Cell Counter (Thermo Fisher Scientific). Viability was 85.8% for the tumor of the gemcitabine-treated group and 83.6% for the saline-treated group.

### ScRNA-seq

Preparation and sequencing of single-cell RNA-seq libraries were performed in our previous study (Pan et al., 2019; Fei et al., 2020). The single-cell suspensions were converted in a Chromium single-cell controller to generate single-cell gel beads in the emulsion, using the Chromium Single-Cell 5′ Library and Gel Bead Kit and Chromium Single-Cell A Chip Kit (10 × Genomics). A total of approximately 10,000 cells to 12,000 cells/chip were captured on the ×10 Chromium platform. The libraries were pair-end sequenced on the Illumina NovaSeq 6000 platform with read lengths of 150 bp (performed by CapitalBio, Beijing). All the procedures, including the complementary DNA synthesis and library preparation, were performed according to the standard manufacturer’s protocol using version 2 chemistry.

### Cell Ranger pipeline

Cell Ranger software version 3.0.1, available from 10x Genomics, was used to process raw sequencing data obtained from the Illumina sequencing output with default and recommended parameters. In short, raw base call files were converted to FASTQ files for each sample by cellranger mkfastq. The FASTQ files were mapped to the GRCh38 human reference genome and mm10 mouse reference genome to distinguish human and mouse cells using cellranger count. Then, feature-barcode matrices were generated for each sample by filtering, barcode counting, and unique molecular identifier (UMI) counting. The cellranger aggr pipeline was used to integrate the data from two samples into an experiment-wide feature-barcode matrix. Finally, the feature-barcode matrix was loaded to the R package Seurat for quality control and downstream analyses.

### Seurat pipeline

The combined dataset was read into the Seurat R package (version 3.5.2) (Satija et al., 2015). First, low-quality cells were filtered out according to the following thresholds.

**Table:** 

Index	Threshold
nUMI	≥1,000
nGene	≥300
log_10_GenesPerUMI	>0.8
Mito.percent	<20%

Using these thresholds, the number of cells varied as follows:

(1) **Raw**: 12C: 1,291,344,765 reads; 12G: 1,218,276,921 reads.(2) **Cellranger**: 12C: 12,868 cells (10,175 human cells and 2,693 mouse cells); 12G: 11,969 cells (8,519 human cells and 3,450 mouse cells). (3) **Thresholds to filter droplets**: 12C: 7,299 cells (5,334 human cells and 1,965 mouse cells); 12G: 6,729 cells (4,446 human cells and 2,283 mouse cells).

Then, the data were normalized and scaled through Seurat’s NormalizeData and ScaleData functions. The highly variable gene (HVG) was identified using the FindVariableGenes function for the next principal component analysis (PCA) with default parameters. PCA was performed based on approximately 2,000 variant genes, and the first 40 PCA components were used for the t-distributed stochastic neighbor embedding (t-SNE) dimension reduction. Cell clusters were identified by running the FindClusters function of Seurat.

### Identification of cluster-specific genes and pathway enrichment analysis

To confirm marker genes, the FindAllMarkers function was used with the MAST test for single-cell gene expression. For each cluster, only genes that were expressed in >25% of cells with at least a 0.25-fold difference were considered. For pathway analysis, the gene sets were downloaded from the Molecular Signature Database (MSigDB), the GSVA R software package was applied to the scRNA-seq data, and pathway scores were calculated for each cell. Pathway enrichment analysis of bulk RNA-seq was performed using the limma R software package.

### Copy number variation analysis

To distinguish tumor cells from all cells, copy number variation (CNV) analysis was performed with R package inferCNV under default parameters (https://github.com/broadinstitute/inferCNV) according to the previous studies. The fibroblasts (cluster 17) were used as reference normal cells for CNV analysis. The CNV score was calculated as a quadratic sum of the CNV region.

### RNA velocity

We performed this analysis as described by La Manno et al., 2018. Based on our aligned bam files of scRNA-seq data, the number of reads mapping to spliced and unspliced transcripts was counted using the velocyto python package. The calculation of RNA velocity values for each gene in each cell and embedding RNA velocity vector to low-dimension space was performed as previously described. All cells used for downstream analysis were taken into velocity models to allow for more accurate estimation of the velocity steady states.

### Construction and validation of the 5-gene risk model

To construct the prognostic model, PDAC patients in The Cancer Genome Atlas (TCGA) dataset were used as the training set. The univariate Cox regression analysis was performed to determine the association between the cluster 10 marker genes identified by scRNA-seq analysis and overall survival (OS). Significant genes related to OS (*p* < 0.05) were selected for further least absolute shrinkage and selection operator (LASSO) regression analysis. Then, LASSO-selected genes were used in the multivariate Cox regression analysis to evaluate the independent prognostic value of each gene. The risk score formula was constructed as described in the previous study (Ma et al., 2018). Patients in the training set were categorized into high-risk and low-risk groups based on the median risk score, which is described in the previous study (Wu and Zhang, 2020; Liu et al., 2021). The OS of the two groups were compared using the Kaplan–Meier (K–M) method, and the time-dependent receiver operating characteristic (ROC) curves were used to evaluate the accuracy of the prognostic model. Multivariate Cox regression analyses were performed to investigate whether the risk score was independent of relevant clinical features. PDAC patients of TCGA treated with gemcitabine were selected, grouped into high- and low-risk groups, and analyzed for complete response (CR) *versus* progressive disease (PD). Finally, GSE71729 and GSE62452 from the Gene Expression Omnibus (GEO) (Moffitt et al., 2015; Yang et al., 2016) and pancreatic cancer-CA (PACA-CA) from the International Cancer Genome Consortium (ICGC) were employed for external validation.

### Statistical analysis

At least three biological replicates were used in each experiment unless otherwise stated. Two-tailed Student’s t-tests and one-way ANOVA were used for analyzing the quantitative data. Statistical significance was defined as a *p*-value <0.05. Prism8 (GraphPad) was used for statistical analyses.

## Results

### PDXs show diverse responses to gemcitabine, and the most notable responder is identified

**FIGURE 1:**
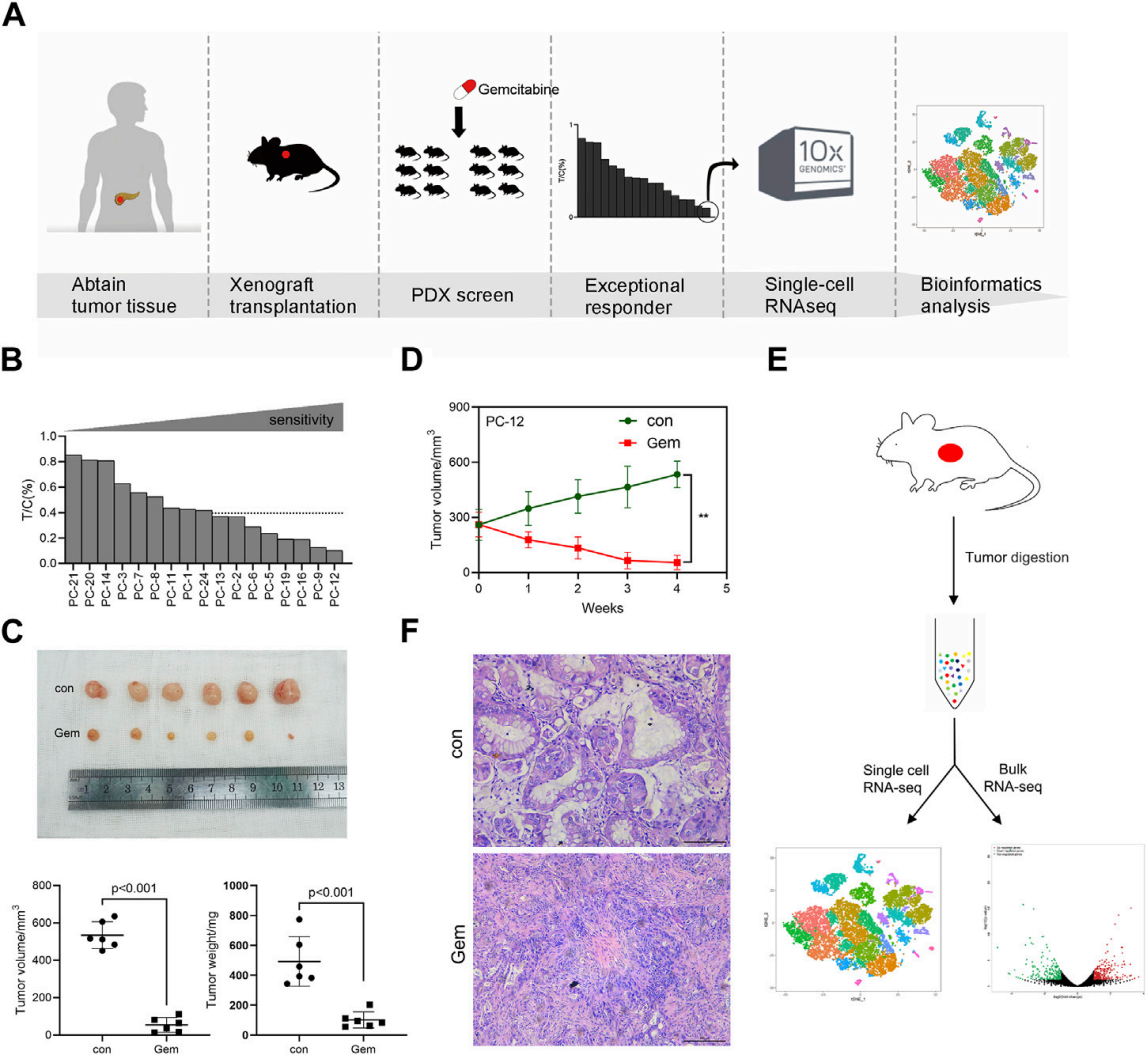
Experimental overview. One PDAC PDX displays notable sensibility to gemcitabine. **(A)** Schematic diagram of experiments. PDXs of PDAC patient tumors were established and propagated by xenograft transplantation in NOD mice for gemcitabine screening. PDXs most sensitive to gemcitabine were screened (PC-12) to perform single-cell RNA-seq. **(B)** Relative tumor growth rate (T/C%) of 17 PDAC PDX models treated with gemcitabine (n = 5–8) *versus* untreated (n = 5–8). **(C)** PC-12 xenografts from NOD mice were treated intraperitoneally with normal saline control or gemcitabine at 100 mg/kg twice per week for 4 weeks. Each group contained six mice. Tumor volume and weight were then measured. **(D)** Growth curve of PC-12 PDX tumors treated with gemcitabine or untreated (n = 6; *p* < 0.001). **(E)** Tissue processing pipeline for single-cell RNA-seq and bulk RNA-seq. **(F)** Representative H&E sections of the PDX in the control group and gem group.

To explore the sensitivity mechanism of gemcitabine in PDAC, we developed a PDX set by using surgically removed PDAC tissue. We selected 17 PDAC PDX models available. These PDX tumors were histologically confirmed as PDAC, which was also consistent with the diagnosis of the corresponding patients’ immunohistochemistry. The PDX set was screened for sensitivity to gemcitabine, and the overall study design is shown in the experimental scheme diagram (Figure 1A). In this study, the PDX set showed diverse responses to gemcitabine, and the relative tumor growth rate (T/C%) ranged from 10% in the most sensitive to 85% in the least sensitive model. Eight of the 17 PDX models showed T/C% less than 40%, and PC-12 showed the most significant tumor growth inhibition in all models (Figure 1B, Supplementary Figure S1A). The notable responder (PC-12) showed remarkable tumor regression after gemcitabine treatment, as assessed by the tumor volume (*p* < 0.001) and weight (*p* < 0.001; Figures 1C, D).

The clinical characteristics and treatment history of patients providing the samples for PDX establishment are listed in Supplementary Table S1. Of the 17 patients, the majority had TNM stage II–III (13 cases) and were moderately differentiated (15 cases). A total of 13 patients underwent postoperative chemotherapy, and neoadjuvant therapy was not performed in any patients in this cohort. Consistent with the clinical benefit of the patients in the set, patients sensitive to gemcitabine in PDX showed better OS (Supplementary Figure S1B, Supplementary Table S1). The most notable responder who received a full course of gemcitabine chemotherapy showed an optimal OS among the 17 patients. The median OS was 13.7 months.

### Single-cell transcriptome profiling of the notable responder

To comprehensively investigate the contribution of heterogeneity for tumor cells to gemcitabine resistance, we performed scRNA-seq and bulk RNA-seq on freshly dissociated cells of non-treatment and post-treatment PDX models for the notable responder (PC-12) (Figure 1E). As reported previously, the human tumor-associated stroma is rapidly lost and replaced by mouse stromal cells in a PDX model (Schneeberger et al., 2016; Knudsen et al., 2018). We used standard methods to obtain single cells from tumor tissues of PDXs for scRNA-seq that consist of human and mouse cells. The scRNA-seq data were compared with the human and mouse reference genomes using a computer algorithm to distinguish human and mouse cells. To unravel the heterogeneity of the tumor, we further clustered cells based on gene expression levels via the Cell Ranger and Seurat R packages. First, the data were subjected to quality control to filter out low-quality cells. The total number of cells retained after stringent quality control filtering was 14,028, comprising 7,299 cells originating from the control group and 6,729 cells originating from the gem group. The control group included 5,334 human cells and 1,965 mouse cells, and the gem group included 4,446 human cells and 2,283 mouse cells. Then, normalization and PCA were performed for dimension reduction. We divided cells into variant clusters with similar gene expression signatures via unsupervised graph clustering, and we used Seurat’s t-SNE to visualize the clusters. We identified two major human cell types in the PDX model: tumor cells and fibroblasts. We also revealed that the tumor microenvironment (TME) was mostly composed of murine cells in the PDX, including fibroblasts, endothelial cells, neutrophils, macrophages, and natural killer (NK) cells. Stromal cells were abundant in the tumor tissues of PDAC, whereas our results showed few fibroblasts in human cells and many murine stromal cells in the PDX model, indicating that the stromal components of human cells were lost and replaced by stromal cells of mice in the PDX model (Figure 1F).

### Intratumoral cellular heterogeneity changes during gemcitabine treatment

**FIGURE 2:**
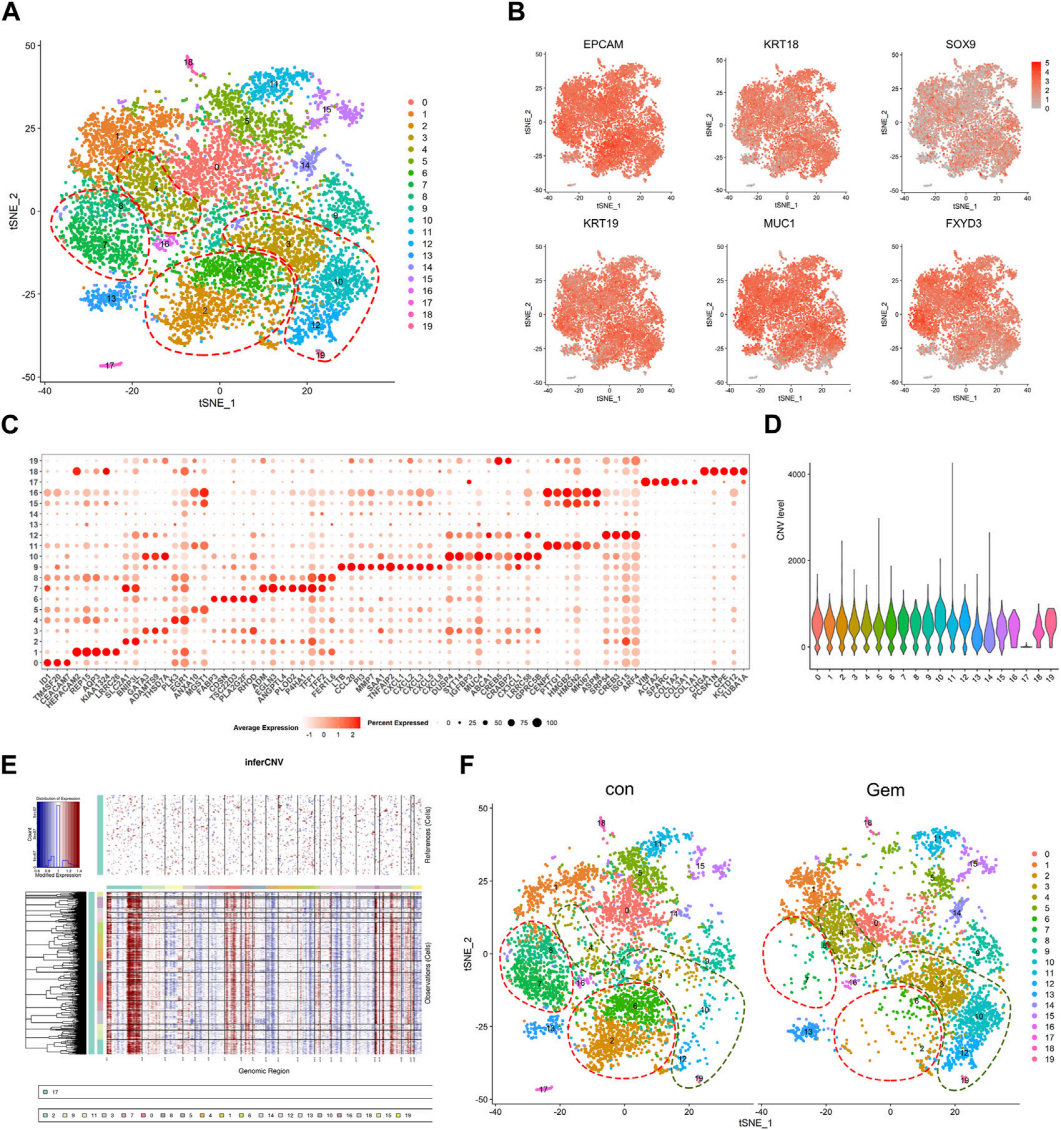
Single-cell analysis reveals cellular heterogeneity between the control group and gem group of human PDAC. **(A)** t-SNE plot of intratumoral cells from the two groups merged. **(B)** t-SNE plot of tumor cells displaying representative marker gene expression. The intensity of color indicates the level of average expression. **(C)** Bubble plot showing selected cell type-specific markers across all clusters. The size of dots represents the fraction of cells expressing a particular marker, and the intensity of color indicates the level of average expression. **(D)** Violin plot showing the CNV score of each subpopulation. **(E)** Heatmap showing the large-scale CNV profile of each cell and reference cell subpopulation; the red and blue colors represent high and low CNV levels, respectively. **(F)** Comparison of each cluster in the two groups.

To investigate the cellular diversity and molecular features, we further divided the human cells into 20 clusters based on the t-SNE analysis (Figure 2A). The gene expression levels were compared, and specific gene sets of each cluster were used to distinguish these clusters. Notably, we found that the ductal cell markers and epithelial cell-specific markers reported previously, such as EPCAM, KRT18, SOX9, KRT19, MUC1, and FXYD3 (Peng et al., 2019; Moncada et al., 2020; Chen et al., 2021), were commonly expressed in the majority of clusters except for cluster 17, confirming tumor cell identity. In contrast, cluster 17 was exclusively observed to have high expression of special fibroblast markers, such as VIM, COL1A1, COL1A2, and SPARC (Bernard et al., 2019; Elyada et al., 2019), suggesting that cluster 17 is a fibroblast in PDAC (Figures 2B, C). To distinguish malignant cells more explicitly, we further calculated the large-scale chromosomal CNV for all cells based on the averaged expression patterns of transcriptomes. The inferCNV clustered heatmap and violin plots were created to demonstrate the distributions of CNV scores among different cell types. We found that the majority of clusters exhibited markedly higher CNV levels than cluster 17, which further supported our clustering (Figures 2D, E).

To reveal the remodeling of tumor cell subsets induced by gemcitabine, cells from two different clusters are shown separately in the t-SNE map, and the corresponding cell numbers of clusters are shown in the histogram (Figures 2F, 4D upper panel). We observed that gemcitabine results in a major redistribution of tumor cell subsets with a substantial reduction in clusters 0, 2, 5, 6, 7, and 8 and an enhanced presence of clusters 3, 4, 10, 12, and 19. This finding indicates that, in the tumors of sensitive individuals, the different subclones also showed varying degrees of gemcitabine sensitivity. Furthermore, we found that several upregulated genes in those enhanced subclones, including SYT14, CREB5, and ABCA1, were associated with drug resistance and tumor proliferation in previous reports (Sheng et al., 2018; Wang et al., 2020; Oberle et al., 2022; Tong et al., 2022) (Figures 2C, 4B). Previous studies have shown that treatment resistance may result from competition in the fitness of pre-existing resistance subclones (Nowell, 1976), which is consistent with our results. In conclusion, our results showed that gemcitabine remodeled intratumoral subclones and promoted the proliferation of resistance subclones.

### Tumor microenvironment remodeling induced by gemcitabine in pancreatic cancer

**FIGURE 3:**
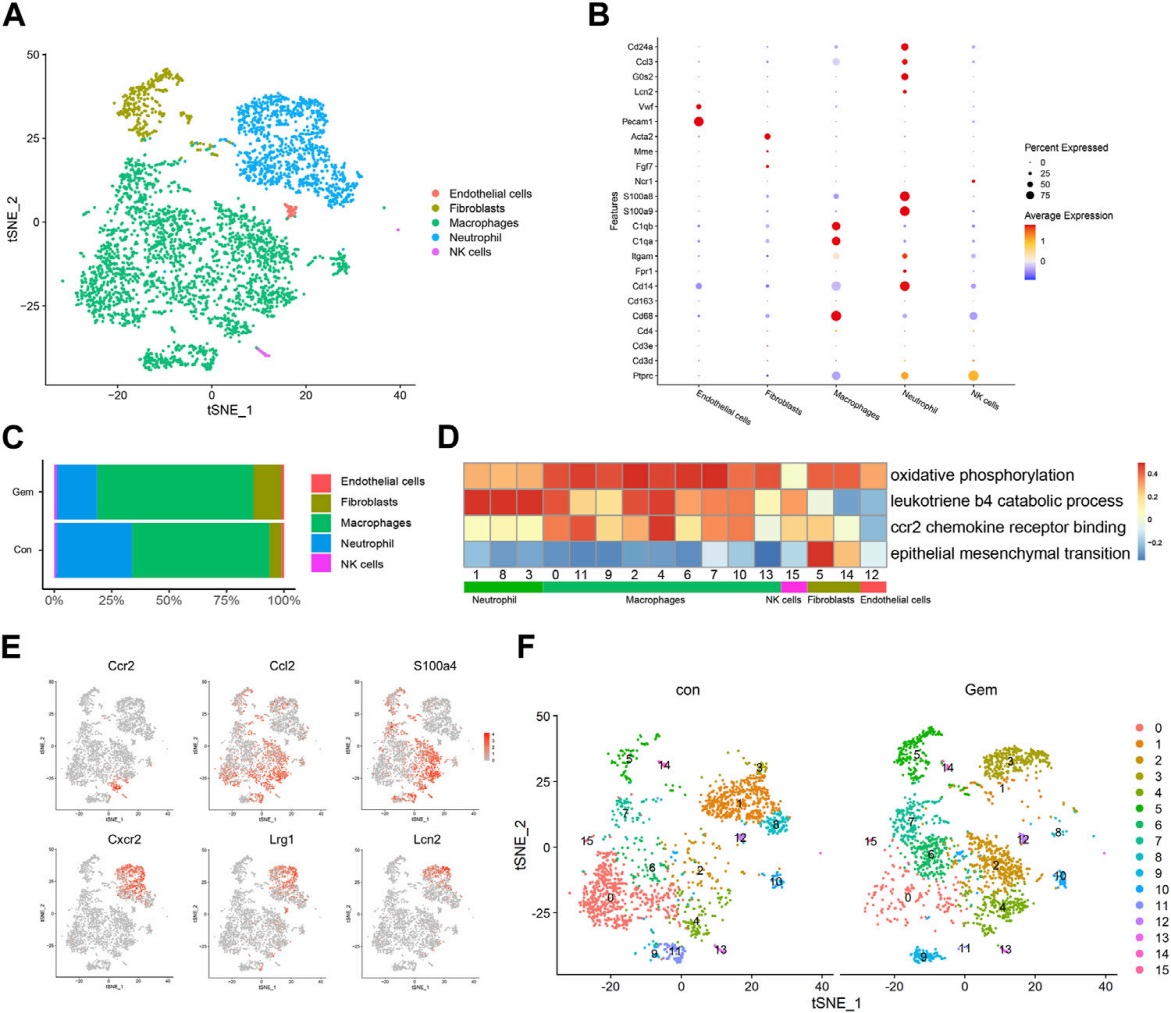
Gemcitabine induces remodeling of tumor-infiltrating lymphoid cells. **(A)** t-SNE plot showing the distribution of 4,248 mouse cells in mixed tumors of the control and gem groups. Cells are colored according to their corresponding cell type. **(B)** Bubble plot showing selected cell type-specific markers of all clusters. The dot size represents the percentage of cells within the subtype, and the intensity of color indicates the level of average expression. **(C)** Proportion of mouse cell clusters from the control group and gem group. **(D)** Heatmap of Hallmark and Gene Ontology pathways enriched in mouse cell clusters. **(E)** t-SNE plots displaying representative marker gene expression of cluster 3 (macrophage) and cluster 4 (neutrophil). The intensity of color indicates the level of average expression. **(F)** Comparison of each cluster in the two groups.

Treatment-induced alterations in the TME also affect tumor chemoresistance and progression. As mentioned earlier, most of the TME in PDX was replaced by murine cells. To investigate the changes in the TME after gemcitabine treatment, we analyzed the scRNA-seq data of murine cells in PDX tumors of the gem and control groups. Cell types were annotated by SingleR and known cell-type markers (Hosein et al., 2019; Fei et al., 2020). Three distinct neutrophil clusters, nine distinct macrophage clusters, one cluster of NK cells, one cluster of fibroblasts, and one cluster of endothelial cells were identified (Figures 3A, B). Our results showed that gemcitabine induced the remodeling of TME cells with a notable reduction in neutrophils and enhancement of the presence of macrophages and fibroblasts.

Tumor-infiltrating macrophages represented the largest proportion in both groups, as shown in Figure 3C, and the total number and proportion increased after gemcitabine treatment. Macrophages showed high levels of oxidative phosphorylation, which is reported to be characteristic of M2 macrophages (Saha et al., 2017; Viola et al., 2019) (Figure 3D). In cluster 4, which mostly existed in the gem group, cells expressed high levels of CCR2, CCL2, and S100A4 genes that are associated with tumor progression and metastasis (Dahlmann et al., 2016; Li et al., 2017) (Figures 3E, F, Supplementary Figures S1D, F). Gene Ontology and KEGG enrichment analyses revealed that cluster 4 displayed upregulation of pathways associated with CCR2 chemokine receptor binding (Figure 3D). Using immunohistochemical staining, we confirmed that the number of CD68^+^ macrophages and CCR2^+^ cells increased after gemcitabine treatment (Supplementary Figures S1C, D, F). We also identified a significant decline in the neutrophil cluster, which expressed genes encoding S100A8, S100A9, CD24a, G0S2, and CCL3 (Figure 3B), from an average of 33.1% in the control group to an average of 17.6% in the gem group (Figure 3C). In addition, the number of LY6G^+^ cells decreased after gemcitabine treatment in the immunohistochemical analysis (Supplementary Figures S1E, F). However, neutrophils in the gem group expressed high levels of CXCR2, LRG1, and LCN2 genes (Figures 3E, F), which are associated with tumor progression and metastasis (Yao et al., 2020; Olson et al., 2021; Singhal et al., 2021). Following gemcitabine treatment, the proportions of fibroblasts increased, and the fibroblasts displayed positive regulation of epithelial–mesenchymal transition (Figures 3C, D).

Although the adaptive immune system of NOD-SCID mice is compromised, studies have shown that functional macrophages and NK cells are still present in NOD-SCID mice (Miao et al., 2021; Zhu et al., 2021), which is consistent with our results. It is known that PDAC patients with less infiltration of macrophages in tumor tissue respond better to gemcitabine therapy (Yang et al., 2020). In summary, these findings demonstrate that gemcitabine increased the number of tumor-infiltrating macrophages and suppressed the infiltration of neutrophils, which may be associated with drug resistance.

### Trajectory analysis identified gemcitabine-tolerant subclones

**FIGURE 4:**
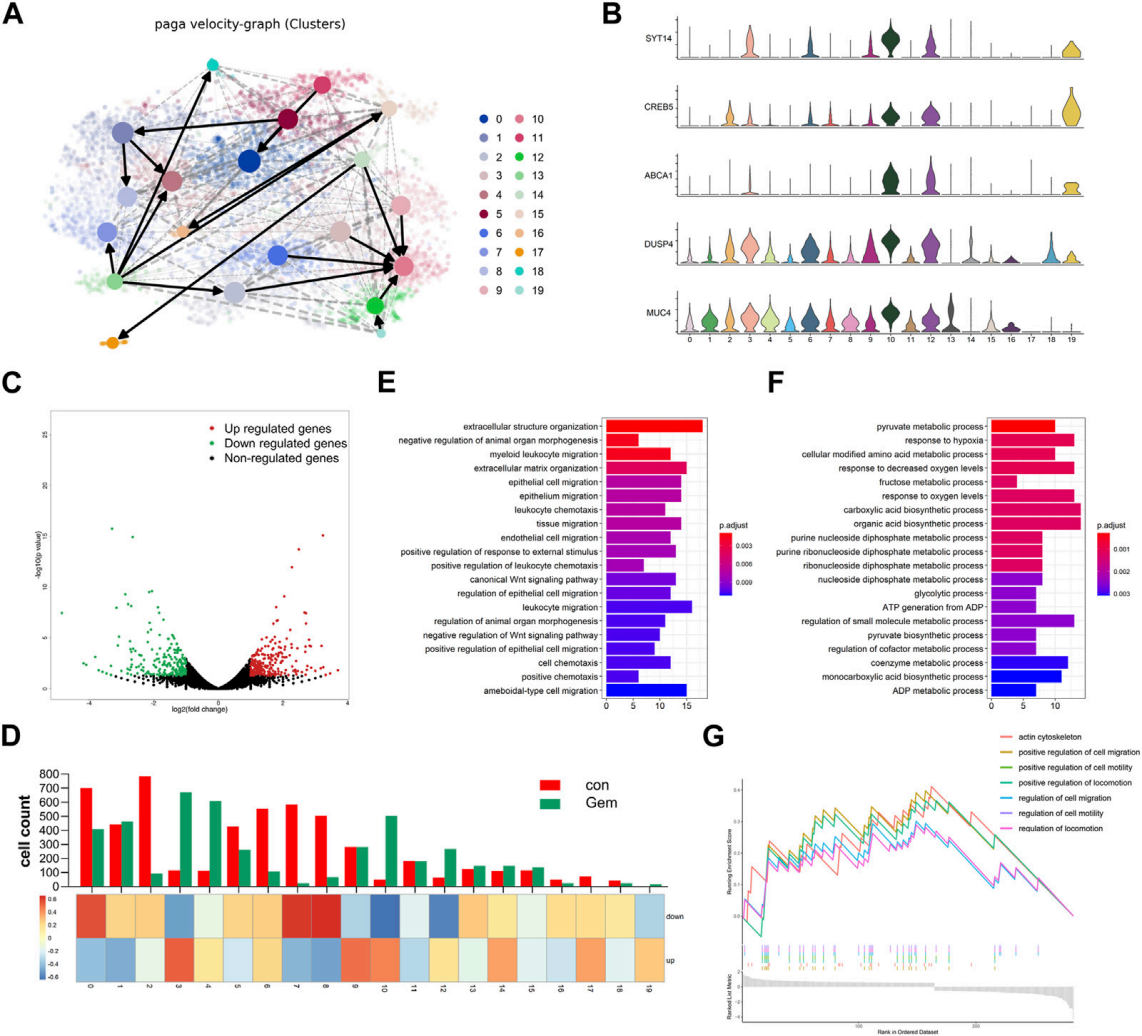
Gemcitabine changed the evolution direction of subclones in tumor cells. **(A)** Velocity graph superimposed to the t-SNE embedding of cells from the two groups merged. Cells are colored according to cell types. **(B)** Violin plots showing the high expression of marker genes of clusters 3, 4, 10, 12, and 19. **(C)** Volcano plot showing the DEGs in bulk RNA-seq data of each group (n = 3). **(D)** The histogram showing the composition of cells in each subpopulation, and the heatmap showing the DEG enrichment in each subpopulation. Bar plots of KEGG enrichment analysis by the upregulated genes **(E)** and the downregulated genes **(F)** of bulk RNA-seq. **(G)** GSEA indicated significant enrichment of GO pathways in the marker genes of cluster 10.

To investigate the evolution among tumor cell populations during gemcitabine treatment, we further performed trajectory analysis. We applied RNA velocity analysis to determine the transcriptional fate of the tumor cells using information about the expression of genes at the unspliced and spliced levels. The data from the gem and control groups were merged, and the projection of the velocity field arrows on the t-SNE plot extrapolated the future state of subclones (Figure 4A, Supplementary Figures S2A, B). Interestingly, velocity graphs showed that there was a common evolutionary direction of clusters 2, 3, 6, 9, 12, 14, and 19, and these paths ultimately converged and terminated in cluster 10 (Figure 4A). Gene expression analysis revealed that the expression of genes associated with tumorigenesis and drug resistance, including SYT14, CREB5, and ABCA1, gradually increased during transformation toward cluster 10 (Figure 4B). This finding demonstrated that cluster 10 may be a special subclone related to gemcitabine tolerance. Gemcitabine results in the remodeling of the subclones of tumor cells in PDAC tumors with a notable reduction in cluster 8 and the enhanced presence of cluster 4. Compared with cluster 8, genes related to tumor proliferation and drug resistance, such as DUSP4 and MUC4 (Xu et al., 2020; Chen et al., 2021), were highly expressed in cluster 4 (Figure 4B). This result indicated that the evolutionary direction of tumor cells was changed by gemcitabine, and tumor cells transformed into resistant populations under the pressure of chemotherapy. Therefore, our results revealed the dynamic differentiation trajectories of PDAC tumor subclones during gemcitabine treatment. Although preliminary, our findings suggest the interesting hypothesis that gemcitabine resistance arises from a small pre-existing resistant subclone that transforms into the major cell type under chemotherapy. Taken together, these data provide compelling evidence for the plasticity of gemcitabine resistance in PDAC.

### Cell type-specific unique gene expression in chemoresistant subclones

To simultaneously define gene expression changes at the global and cellular levels, we also performed bulk RNA-seq of gemcitabine-treated and control samples of the notable responder in parallel. As shown in the volcano map of bulk RNA-seq, 248 upregulated genes and 223 downregulated genes were detected in gemcitabine-treated samples (Figure 4C). Bulk and single-cell transcriptome data were combined to investigate the consistency between single-cell sequencing and bulk RNA-seq. The expression levels of the differentially expressed genes (DEGs) obtained by bulk RNA-seq are displayed as a heatmap of multiple cell types (Figure 4D, Supplementary Figures S2C, D). Most of the upregulated genes were concentrated in clusters 3, 9, and 10, and most of the downregulated genes were concentrated in clusters 0, 7, and 8. The cell count of cluster 9 was shown to be unaltered in the scRNA-seq data of the control and gem groups, whereas it was obviously enriched in upregulated genes of bulk RNA-seq (Figure 4D). Conversely, clusters 2, 5, and 6 were downregulated markedly in the gem group, whereas the downregulated genes were not enriched in these subgroups. Taken together, these differences reflect the intercellular heterogeneity of gene expression, suggesting that further investigation of gene expression diversity in each cell type of tumor cells in PDAC is important.

To further investigate the biological function of these genes, functional enrichment analysis was performed on the DEGs. The results indicated that extracellular structure organization and epithelial cell migration were specifically activated in the upregulated genes of the gem group, whereas the downregulated genes were mainly enriched in the pyruvate metabolic process and response to hypoxia (Figures 4E, F). To identify characteristics in the resistant subgroup, we further performed GSEA with the marker genes of cluster 10, which was aforementioned as a terminal and typical resistant subgroup. Cluster 10 marker genes were highly enriched in the regulation of cell migration pathways, pathways regulating cell motility, and pathways regulating locomotion (Figure 4G), which were associated with tumor metastasis and proliferation.

### Development of the transcriptional prognostic signature

**FIGURE 5:**
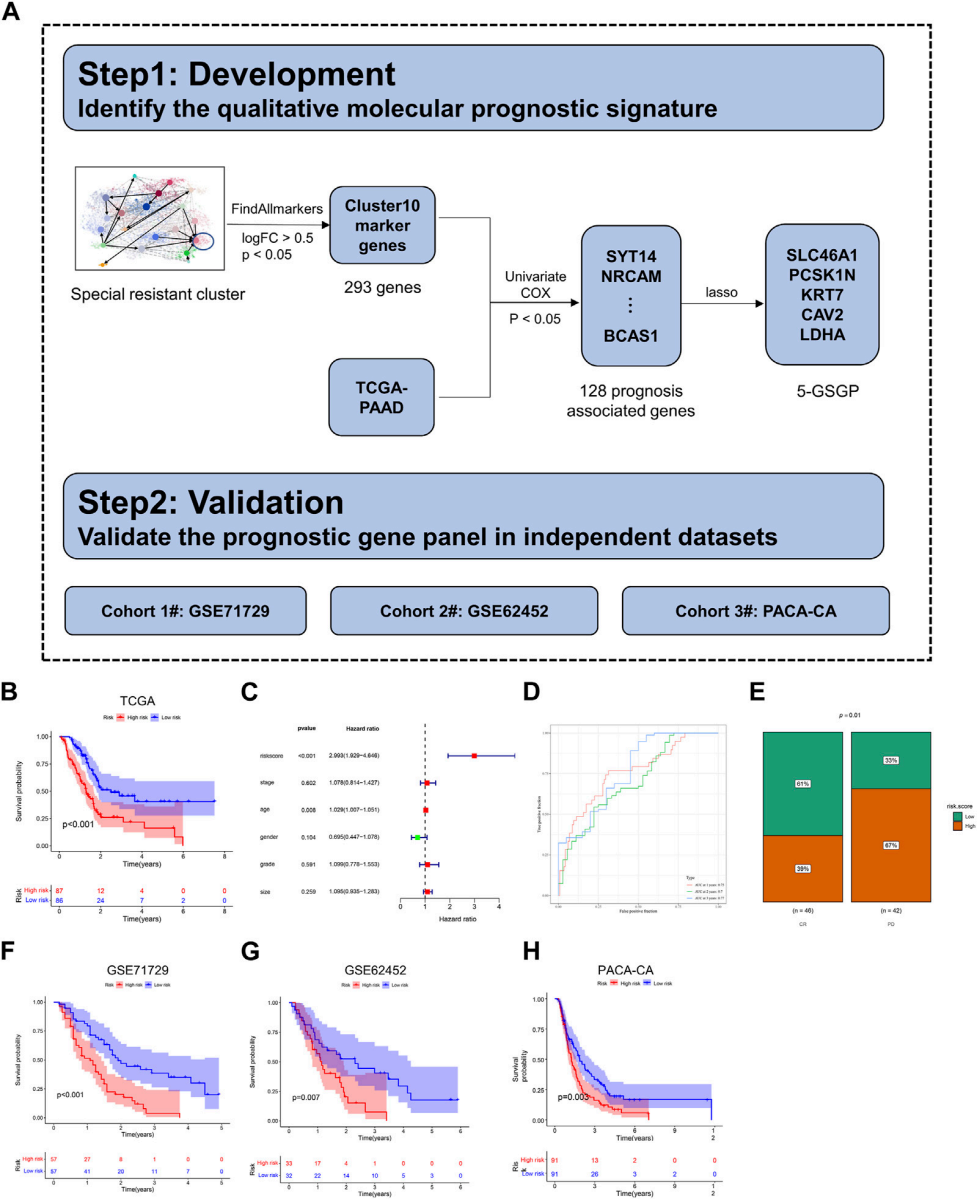
Construction of a diagnostic model. **(A)** The flowchart for development and validation of the diagnostic model. **(B)** The K–M curves of OS for the two prognostic groups stratified by the risk score in the training cohort. **(C)** Univariate Cox regression analysis of clinical characteristics and the risk score. **(D)** Comparison of time-dependent ROC curves of the risk score for predicting the 1-, 2-, and 3-year OS rates. **(E)** Hierarchy graph demonstrating TCGA analysis on high- and low-risk scores of PDAC patients with progressive disease (PD) and complete response (CR) after gemcitabine treatment. **(F–H)** The K–M curves of OS for the two prognostic groups stratified by the risk score in the validation cohorts.

The general flowchart of this part is described in Figure 5A. Based on the trajectory and gene enrichment results, we found that cluster 10 was a specific subclone of drug resistance. To investigate the gene expression characteristics of the drug-resistant subclone of PDAC, we analyzed the transcriptional data of cluster 10. First, 293 marker genes of cluster 10 were identified (log_2_FC > 0.5, *p* < 0.05, Supplementary Table S2) by the function FindAllmarkers. Then, 128 genes predicting the prognosis of PDAC were preliminarily screened out among the 293 marker genes using univariate Cox’s proportional hazard model (*p* < 0.05, Supplementary Table S3) with mRNA expression data in the TCGA dataset (n = 173). Next, LASSO regression analysis was performed for these genes to further eliminate confounders (Supplementary Figures S3A–C). According to the results of LASSO regression, five genes (SLC46A1, PCSK1N, KRT7, CAV2, and LDHA) were selected as a 5-GSGP to construct the prognostic prediction model. According to the model, we calculated the risk scores of 173 PDAC patients in the TCGA dataset, and patients were divided into high- and low-risk groups based on the median value. K–M analysis showed that the low-risk PDAC patients were significantly associated with a favorable OS (*p* < 0.001, Figure 5B). Multivariate Cox regression analysis showed that the 5-GSGP was an independent risk factor associated with patient OS (HR = 2.993%95% CI: 1.929–4.646, *p* < 0.001, Figure 5C) after adjusting for stage, tumor size, grade, age, and sex in the TCGA dataset. Then, the areas under the ROC curves (AUCs) were calculated to assess the OS prediction efficiency of the risk model (AUC_1yr_ = 0.75, AUC_2yr_ = 0.7, and AUC_3yr_ = 0.77, Figure 5D), indicating that this model had favorable predictive power. In addition, to understand the clinical association of the risk score with PDAC chemoresistance, patients who received gemcitabine in the TCGA dataset were segregated into high- and low-risk groups by the median value. Low-risk patients were more responsive to gemcitabine, and 67% of patients with a high-risk score exhibited progressive disease after gemcitabine treatment (*p* = 0.01, Figure 5E).

To validate the prognostic value of the 5-GSGP, we further calculated the risk scores of PDAC patients in GSE71729, GSE62452, and PACA-CA. Notably, the risk scores divided the patients of validation sets into high-risk and low-risk subgroups, and the high-risk group had significantly shorter OS than the low-risk group (*p* < 0.001, *p* = 0.007, and *p* = 0.003, Figures 5F–H). We further calculated the AUCs of the three validation cohorts (AUC_1yr_ = 0.68, AUC_2yr_ = 0.72, AUC_3yr_ = 0.81; AUC_1yr_ = 0.62, AUC_2yr_ = 0.77, AUC_3yr_ = 0.82; and AUC_1yr_ = 0.59, AUC_2yr_ = 0.66, and AUC_3yr_ = 0.67; Supplementary Figures S3D–F), and the risk score also showed a favorable predictive power. The results showed that the 5-GSGP can robustly predict the prognosis of PDAC patients both in the TCGA dataset and in independent validation datasets.

## Discussion

PDAC is highly malignant with extremely poor outcomes. As we know, chemoresistance is one of the major problems leading to postoperative recurrence and poor overall survival of PDAC. Previous studies have suggested that the cause of drug resistance in PDAC may be pre-existing subsets of drug-resistant cells in tumors (Seth et al., 2019). However, the role of intratumoral heterogeneity and the subclonal architecture in gemcitabine resistance in PDAC patients is unknown. Previous studies on gemcitabine in PDAC have focused on molecular functions and pathways related to drug metabolism, while few studies have been conducted at the level of cellular heterogeneity. In this study, we used scRNA-seq to analyze intratumor heterogeneity in PDAC. Leveraging the PDXs of PDAC, we demonstrated that a small number of subpopulations of tumor cells resistant to gemcitabine already existed in tumors before treatment.

Recently, scRNA-seq has greatly improved our depth of investigation of tumor heterogeneity and even identified the role of rare cell populations in tumor evolution. ScRNA-seq has been used to study chemotherapy resistance in many cancers. According to previous reports, the occurrence of drug resistance in tumors may be due to pre-existing drug resistance subclones in the original cell population. Aissa et al. revealed that scRNA-seq can be used to distinguish drug-tolerant states and to discover unique drug-resistant cell subpopulations (Aissa et al., 2021). Kim et al. identified that subpopulations exhibit differential therapeutic sensitivity by scRNA-seq in lung cancer (Kim et al., 2015). In addition, Savage et al. revealed that EGFR^high^ subpopulations in triple-negative breast cancer tumors showed particular sensitivity to gefitinib (Savage et al., 2017). Our scRNA-seq data identified a specific drug-resistant subpopulation in tumors that existed prior to gemcitabine treatment, survived, and proliferated to become the dominant subpopulation. We further investigated the particular drug-resistant subpopulation and established a 5-GSGP for PDAC patients. The 5-GSGP was an independent risk factor for PDAC and could be used to assess survival and to predict the sensitivity to gemcitabine of PDAC patients.

Previous studies have explored the impact of chemotherapy on tumor evolution, while the effect in PDAC remains unclear. Nowell et al. first proposed the theory of clonal evolution of tumor cell populations, which states that tumor progression results from successive rounds of clone selection (Nowell, 1976). Maynard et al. (2020) revealed the therapy-induced evolution of human lung cancer. Our results also found that chemotherapy changed the predominant subpopulations in tumors, which led to the evolution of subpopulations toward drug resistance. As a result of adaptive competition among tumor cell subpopulations under pharmacological pressure, tumors are able to develop drug resistance. The function and mechanism of therapy-induced evolution in PDAC warrant further investigation.

Our data suggest that there may likely be complex relationships between molecular alterations and the subpopulations that exist in PDAC tumors treated with gemcitabine. We identified significantly altered genes after gemcitabine treatment that are potentially associated with resistance. Candidate resistance genes from our work include DUSP4, ABCA1, and SYT14. DUSP4, a member of the dual specificity phosphatase family, is considered an oncogenic gene that has been shown to be associated with proliferation, migration, and tumorigenicity in esophageal squamous cell carcinoma, renal cell carcinoma, and colorectal cancer (Xu et al., 2020; Han et al., 2021; Zeng et al., 2021). In addition, DUSP4 expression was significantly correlated with the prognosis of PDAC patients in the TCGA database. The ABCA1 gene, which has not been studied in depth in PDAC, is a member of the ABC transporter family. ABCA1 has been shown to play a crucial role in the development of resistance and proliferation of colorectal cancer (Aguirre-Portoles et al., 2018). Furthermore, increased expression of ABCA1 is associated with the development of acquired chemotherapy resistance and poor patient outcome in ovarian cancer (Wang et al., 2021; Gao et al., 2022). SYT14 is a membrane-trafficking protein that can promote the growth of human glioma cells (Sheng et al., 2018).

The TME has been suggested to play a key role in the chemoresistance of many cancers, including PDAC, and it has been reported that increased tumor-infiltrating macrophages are associated with therapeutic resistance (Bulle et al., 2020; Minz et al., 2022). In our study, we found that gemcitabine promoted the infiltration of macrophages and recruited macrophages associated with tumor progression and metastasis. Similar to tumor-infiltrating macrophages, several previous studies have shown that TME-derived neutrophils also play a chemoresistant role. Nielsen et al. reported that suppression of neutrophil development and migration attenuates PDAC progression (Nielsen et al., 2021). In contrast, our results show that gemcitabine reduced the infiltration of neutrophils, whereas, at the same time, it promoted neutrophil expression of genes associated with tumor progression and metastasis. Our study implies that the response induced by gemcitabine in the TME may contribute to the development of drug resistance in PDAC. However, due to the PDX model, where mice are immunocompromised and thus may not accurately represent changes in the microenvironment, changes in other immune cells after gemcitabine treatment warrant further investigation.

The limitations of our study include the small number of PDXs that were analyzed at single-cell resolution. Future work will need to be performed in a larger cohort of PDAC patients to investigate the generalizability of chemotherapy-induced adaptation patterns. Functional studies will also be needed to validate the changes in molecular features after chemotherapy.

## Conclusion

In conclusion, our study provides new insight into the natural selection of tumor cell subclones and the remodeling of TME cells induced by gemcitabine. We revealed a specific drug resistance subclone, and based on the characteristics of this subclone, we constructed a GSGP that can robustly predict gemcitabine sensitivity and prognosis in pancreatic cancer, which provides a theoretical basis for individualized clinical treatment.

## Data Availability

The datasets presented in this study can be found in online repositories. The names of the repository/repositories and accession number(s) can be found in the article/Supplementary Material. ScRNA-seq data on the study are available in the SRA repository, accession number PRJNA943436 (https://www.ncbi.nlm.nih.gov/sra/PRJNA943436) and main R code for the analysis and plotting are available at GitHub (https://github.com/Zelinou/Gem.git).

## Supplementary material

SUPPLEMENTARY FIGURE S1
**(A)** Tumor growth curves for 17 PDX models either left normal saline or gemcitabine. **(B)** The K–M plot of OS in 17 PDAC patients with T/C% less than 40% or T/C% more than 40%. **(C–E)** Results of immunohistochemical staining. **(F)** IHC staining with anti-CD68, anti-CCR2, and anti-LY6G antibodies in the control group and gem group tumors (n = 5) at low (×100) and high magnification (×400). Scale bar = 50 μm (red line at the bottom left).

SUPPLEMENTARY FIGURE S2
**(A–B)** RNA velocity vectors atop are visualized on the t-SNE projection of cells from the two groups merged. **(C–D)** Heatmap showing the DEG enrichment in each subpopulation separately.

SUPPLEMENTARY FIGURE S3
**(A)** Cross-validation for tuning parameter (lambda) screening in the LASSO regression model. **(B)** LASSO coefficient profiles of five genes selected as a 5-GSGP. **(C)** Forest plot of the 128 genes. **(D–F)** Comparison of time-dependent ROC curves of the risk score for predicting the 1-, 2-, and 3-year OS rates in the validation cohorts.
