# Supplementary figures and images for: Single-cell RNA sequencing revealed subclonal heterogeneity and gene signatures of gemcitabine sensitivity in pancreatic cancer
Source: Front Pharmacol. 2023 Jun 1;14:1193791. doi: 10.3389/fphar.2023.1193791 (PMC10267405; doi:10.3389/fphar.2023.1193791)

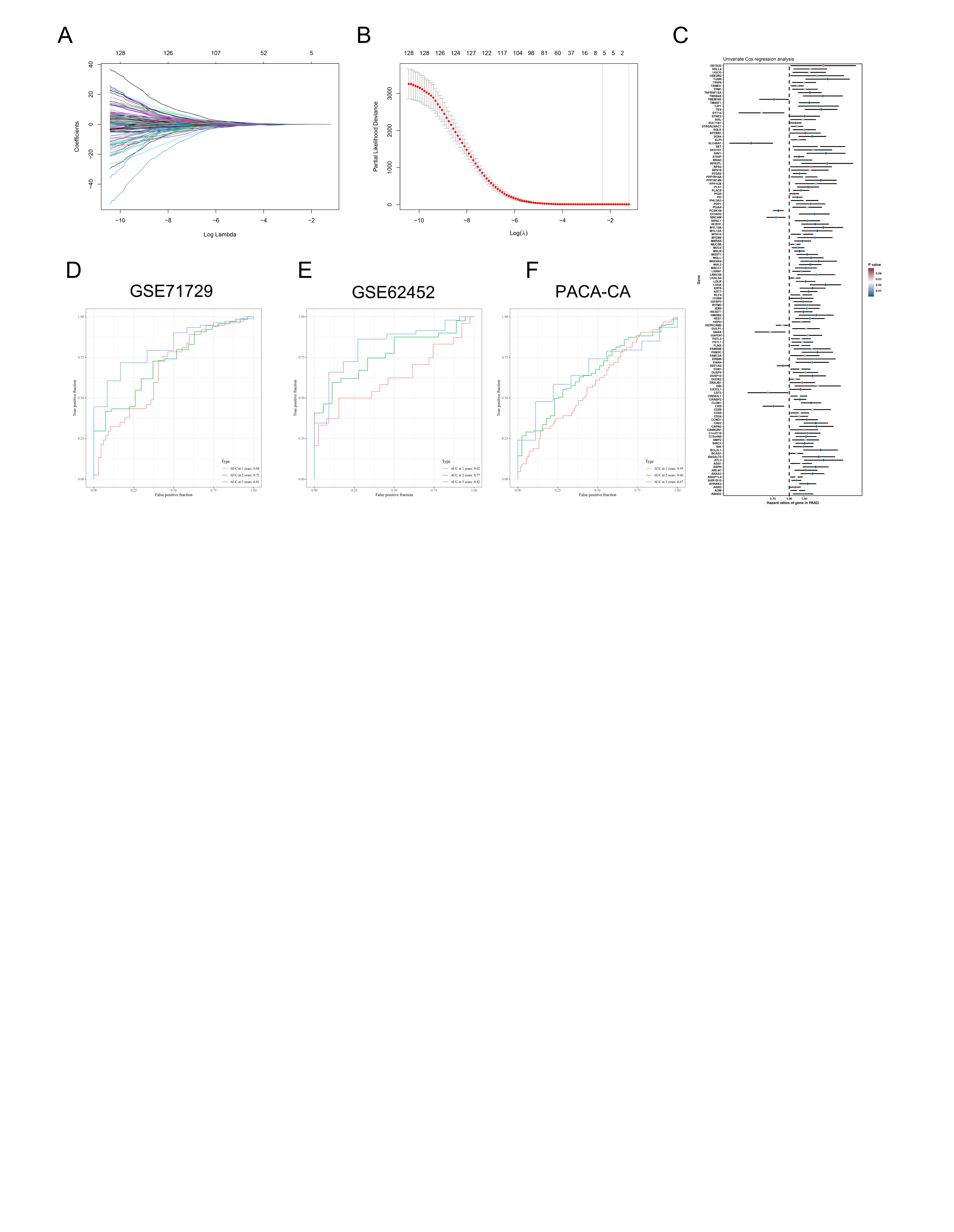

Supplement: Supplementary file 3 [file Image3.TIF]

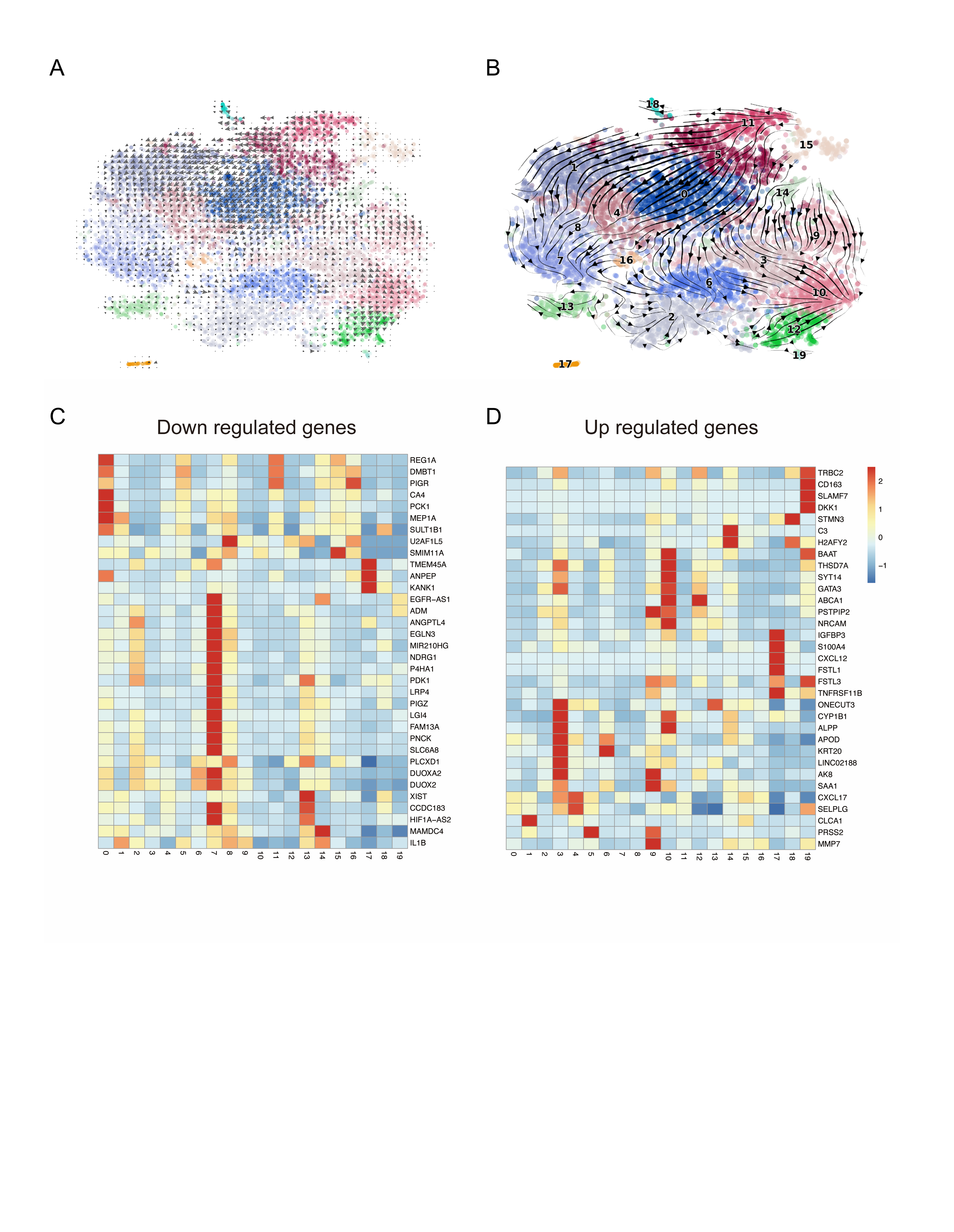

Supplement: Supplementary file 4 [file Image2.TIF]

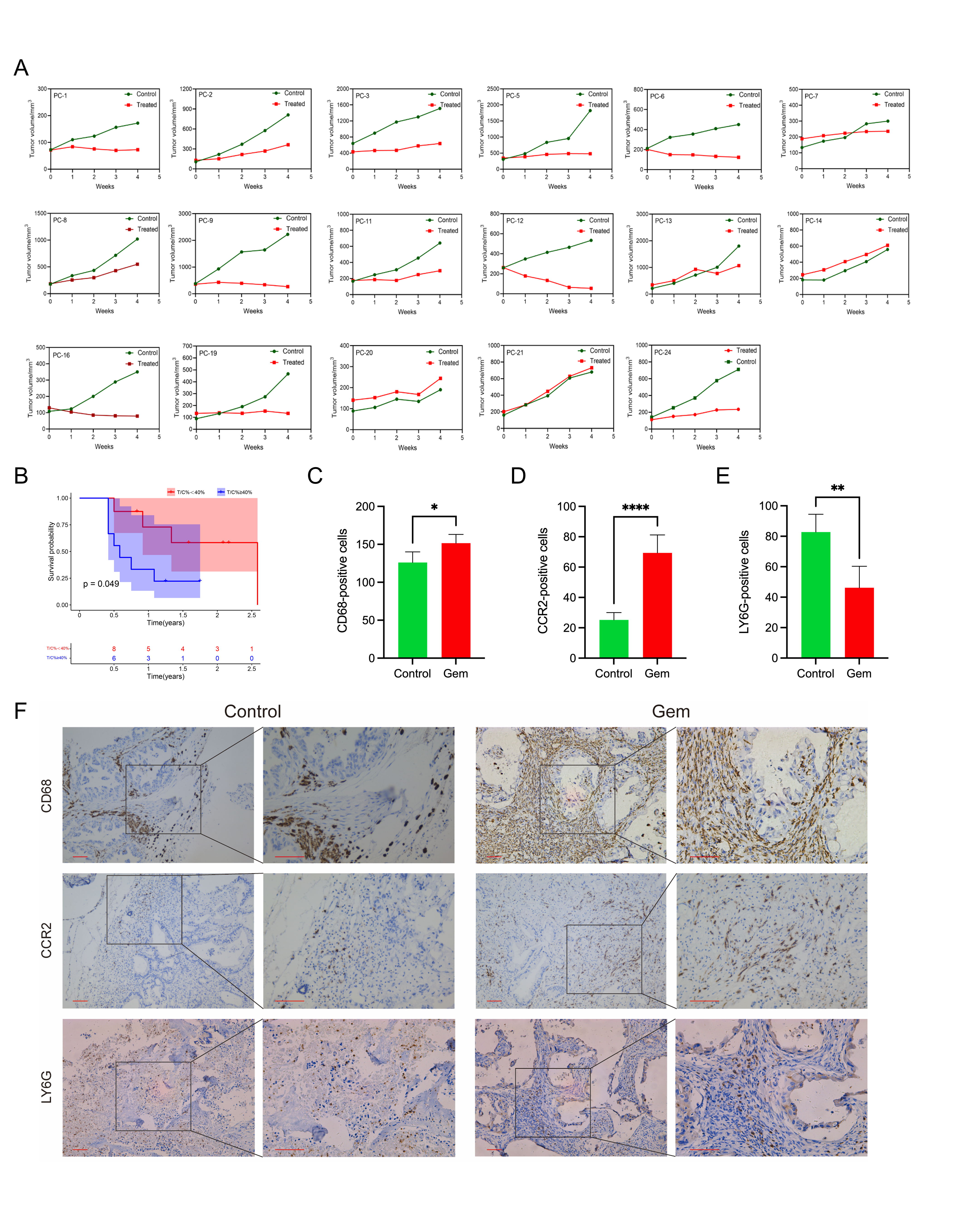

Supplement: Supplementary file 5 [file Image1.TIF]
